# Overexpression of PRDM5 promotes acute myeloid leukemia cell proliferation and migration by activating the JNK pathway

**DOI:** 10.1002/cam4.2261

**Published:** 2019-05-23

**Authors:** Pan Zhou, Xing Chen, Mengke Li, Xiaolu Sun, Jiaqi Tan, Xiaomin Wang, Yajing Chu, Yicheng Zhang, Tao Cheng, Jianfeng Zhou, Gaoxiang Wang, Weiping Yuan

**Affiliations:** ^1^ Department of Hematology Tongji Hospital, Tongji Medical College, Huazhong University of Science and Technology Wuhan Hubei China; ^2^ State Key Laboratory of Experimental Hematology, Institute of Hematology and Blood Diseases Hospital, Department of Stem Cell and Regenerative Medicine Chinese Academy of Medical Sciences and Peking Union Medical College Tianjin China

**Keywords:** acute myeloid leukemia, c‐Myc, JNK, migration, PRDM5, proliferation

## Abstract

PRDM family proteins are dysregulated in many human diseases, especially hematological malignancies and solid cancers, and share a unique N‐terminal PR domain followed by zinc fingers toward the C terminus. With a high frequency of DNA promoter hypermethylation, PRDM5 is primarily considered as a tumor suppressor in solid tumors. However, little is known about the function of PRDM5 in blood malignancies, especially acute myeloid leukemia (AML). In this study, we showed that high PRDM5 expression levels were independently correlated with poor overall survival in AML patients. PRDM5 overexpression promoted cell proliferation, colony formation, and migration in vitro and enhanced tumorigenesis in an in vivo xenograft model. Furthermore, we found that PRDM5 overexpression promoted cell cycle progression with the decreased level of cell cycle inhibitors such as p16 and p21, and regulated the expression of epithelial‐mesenchymal transition markers ZO‐1 and Vimentin to promote migration. Moreover, we observed that PRDM5 upregulated the Jun N‐terminal kinase (JNK) signaling pathway and downregulated c‐Myc expression. Pharmacological inhibition of JNK by SP600125 partially abrogated PRDM5‐induced cell proliferation and migration. Taken together, our findings demonstrate that PRDM5 functions as an oncogenic driver in AML via JNK pathway, suggesting that PRDM5 is a potential therapeutic target for AML.

## 1 INTRODUCTION

Acute myeloid leukemia (AML), the most common blood tumor, is a heterogeneous clonal disorder characterized by the acquisition of different genetic mutations and/or epigenetic changes, the uncontrolled proliferation and blocked apoptosis in immature myeloid progenitors.^1^ Despite recent advances in anticancer and targeted drug therapies, the prognosis of AML is still poor and the relapse rate is high due to an incomplete understanding of the translational and epigenetic regulation of leukemia progression.^2^

The PRDM proteins belong to the SET domain protein family characterized PR domains and zinc fingers, and appear to be transcriptional regulators that either exert enzymatic activity on histones or recruit cofactors to modify target gene expression.^3^ PRDM family members are dysregulated in many human diseases, especially in hematological malignancies and solid tumors, acting as tumor suppressors or drivers during the oncogenic processes.^4^ For example, in a subset of diffuse large B cell lymphoma, PRDM1 promotes cancer progression and is associated with shorter failure‐free survival, while PRDM11 inhibits lymphomagenesis. PRDM3 (MDS1‐EVI1) is critical for the initiation and progression of MLL‐AF9‐induced AML. In prostatic cancers, PRDM16S (MEL1S) is overexpressed and has an antiapoptotic function.^5^, ^6^, ^7^, ^8^, ^9^

PRDM5 (or PFM2) is a recently identified member of the PRDM protein family and is located at human chromosome region 4q26, which harbors many tumor suppressor genes active in several cancers.^10^ PRDM5 participates in inducing committed murine blood cells to differentiate into hematopoietic stem cells and modulates the transcription of a subset of developmental regulators during mouse embryonic stem cell differentiation.^11^, ^12^ Furthermore, PRDM5 silencing by promoter methylation and downregulation has been observed in various types of human solid tumors.^13^ Additionally, several studies have demonstrated that PRDM5 overexpression could induce cell cycle arrest and apoptosis in cancer cells.^10^ Moreover, Wang et al found that PRDM5 overexpression significantly promotes the proliferation and invasion of murine melanoma cells.^14^ These findings indicate that PRDM5 is involved in the regulation of hematopoiesis, cell differentiation, cell cycle, and apoptosis. More importantly, PRDM5 may also be an antioncogene or protooncogene in different types of cancer.

A recent study in melanoma suggests that suppressing JNK blocks PRDM5‐induced tumor progression.^14^ The JNK proteins (Jnk1, Jnk2, and Jnk3) are members of the mitogen‐activated protein kinase (MAPK) family and have been shown to exert controversial effects, acting either to mediate apoptosis or to sustain cell differentiation, proliferation, and tumorigenesis.^15^ Moreover, the JNK signaling pathway has been widely implicated in cancer migration and metastasis. JNK inhibition mediated by the inhibitor SP600125 or specific siRNA impedes the growth of head and neck squamous cell carcinoma, melanoma, and myeloid leukemia.^14^, ^16^, ^17^

Since little is known about the functional role of PRDM5 in hematological malignancies, especially in AML, we investigated the role of PRDM5 in AML cell lines and identified PRDM5 as a tumor promoter in AML. We demonstrated that PRDM5 overexpression promoted the proliferation and migration of OCI‐AML3 and U937 cells in vitro. We further confirmed that the overexpression of PRDM5 in OCI‐AML3 cells and U937 cells promoted tumor proliferation in in vivo xenograft models. In addition, we demonstrated that PRDM5 potentiated cell cycle progression in AML cell lines in a JNK‐dependent manner.

## 2 MATERIALS AND METHODS

### 2.1 Cell culture and healthy donors

Human AML cell lines (OCI‐AML3 and U937) were purchased from the American Type Culture Collection (ATCC, Rockville, MD, USA) and maintained in RPMI‐1640 medium supplemented with 10% fetal bovine serum (FBS). HEK293T cells were cultured in Dulbecco's modified Eagle's medium (DMEM) supplemented with 10% FBS. All cells were incubated at 37°C in humidified air in a 5% CO_2_ incubator. The cell number was counted by the trypan blue dye exclusion method. Normal peripheral blood samples were collected from 3 healthy volunteer donors, and peripheral blood mononuclear cells (PBMCs) were isolated using Ficoll gradient centrifugation. In accordance with the Declaration of Helsinki, all donors signed appropriate informed consent forms before specimen collection and all research involving human subjects was approved by the Ethics Committees of Tongji Hospital.

### 2.2 Antibodies and reagents

Primary antibodies against GAPDH, p‐JNK, p16, ZO‐1, and Vimentin were obtained from Cell Signaling Technologies (Beverly, MA, USA). Anti‐Pan‐JNK, anti‐c‐Myc, and p21 antibodies were purchased from Abcam (Cambridge, MA, USA). The anti‐PRDM5 antibody was purchased from NOVUS Biologicals (Littleton, CO). Secondary goat anti‐mouse and goat anti‐rabbit antibodies were purchased from Cell Signaling Technologies (Beverly, MA, USA). Hoechst 33342 was obtained from Sigma (St Louis, MO, USA). Protease inhibitor cocktail was purchased from Roche Applied Science (Indianapolis, IN, USA). SP600125 (MedChem Express, New Jersey, USA), was dissolved, and stored according to the provided instructions. The SP600125 stock solution was further diluted to the appropriate concentrations in cell culture medium before use.

### 2.3 Expression and survival analysis of data from public datasets

#### 2.3.1 Oncomine database analysis

The Oncomine database (https://www.oncomine.org/resource/login.html), an online database consisting of previously published and open‐access microarray data, was used to analyze the mRNA expression levels of PRDM5 in human AML cells. The fold change in PRDM5 transcription in cells from clinical cancer specimens compared to that in normal control cells was determined using Student's *t* test to generate a *P* value.^18^

#### 2.3.2 SurvExpress database analysis

Data were analyzed from the AML GSE12417‐GPL96 dataset generated by Metzeler and Buske AML GSE12417‐GPL96 from the SurvExpress database (http://bioinformatica.mty.itesm.mx:8080/Biomatec/Survivax.jsp). This validation tool was used for risk estimation using a list of biomarker genes of interest as input for Cox proportional hazards regression.^19^

#### 2.3.3 PrognoScan database analysis

The correlation between PRDM5 mRNA expression and overall survival (OS) was predicted using the PrognoScan database (http://www.abren.net/PrognoScan/). This database is a comprehensive online platform for assessing potential tumor biomarkers and therapeutic targets. To evaluate the OS of patients with AML, patient samples were divided into two groups by median expression (high vs low expression) and analyzed using PrognoScan.^20^

### 2.4 Plasmid construction

Human PRDM5 cDNA was cloned first by RT‐PCR amplification of hPRDM5 mRNA isolated from human PBMCs. The following specific primers were used for amplification: 5′‐CCGGAATTCATGCTGGGCATGTACGTGCCGGACAGGT‐3′ (forward) and 5′‐CGCGGATCCTTAGCTGTCAGCTACACCATGGATATTG‐3′ (reverse). The PCR product was subcloned into the pEasy‐Blunt Zero cloning vector (TransGen Biotech) to generate pEasy‐PRDM5. The construction of pEasy‐PRDM5 was validated by DNA sequencing and this vector served as a template for the construction of the eukaryotic expression plasmids. Ultimately, human PRDM5 cDNA was cloned into the EcoRI/BamHI site of the lentiviral vector pCDH‐MSCV‐EF1‐mCherry (Addgene).

### 2.5 Lentiviral particle packaging and lentiviral infection

HEK293T cells were transfected with an expression vector containing either pCDH‐PRDM5‐mCherry or pCDH‐Migr1‐mCherry and the two packaging plasmids, psPAX2 and pMD2.G, at a mass ratio of 7:5:3, respectively, using Lipofectamine 2000 (Life Technologies, Gaithersburg, MD). Cell culture supernatants were collected at 48 and 72 hours after transfection. The virus particles were passed through a 0.45 μm filter and stored at 4°C.

Human AML cells were transduced with Migr1‐mCherry and PRDM5‐mCherry by two rounds of spinoculation (90 minutes at 1800 rpm) and mCherry‐positive cells were purified by cell sorting using a cell sorter (BD FACS Aria III BD Biosciences).

### 2.6 Cell proliferation

Cells were seeded in 96‐well plates at a density of 5 × 10^3^ cells/well and cell growth was measured by counting viable cells for 6 consecutive days. The in vitro effects of drugs on leukemia cell viability were assessed using a Cell Counting Kit‐8 (CCK‐8, Dojindo Molecular Technologies, Japan) assay according to the manufacturer's instructions. Cells (10000 cells in 100 μL per well) were seeded into 96‐well plates in triplicate and incubated with SP600125 (10, 20, 30, or 40 μmol/L) or vehicle (DMSO) as a control. The absorbance was measured 24 hours later at a wavelength of 450 nm after incubation with CCK‐8 solution at 37°C for 4 hours.

### 2.7 Colony formation assay

Human AML cell colony formation assays were performed in MethoCult H4230 medium (STEMCELL Technologies, Vancouver, CA) at a starting density of 2000 cells/mL. The suspension was dispensed into 24‐well plates at 0.4 mL per well in quadruplicate. Colonies containing more than 20 cells were counted using an inverted microscope after 7‐14 days of culture at 37°C.

### 2.8 Transwell assay

An 8‐μm pore size Costar transwell plate (Corning, Cambridge, MA, USA) was used to measure the migratory potential of OCI‐AML3 and U937 cells. A total of 2 × 10^5^ cells were washed, resuspended in 100 μL of RPMI 1640 medium and seeded in the upper chamber. For the SP600125 rescue experiment, cells were preincubated in 100 μL of RPMI 1640 medium containing 20 μmol/L SP600125 or vehicle control (DMSO) for 2 hours. Subsequently, cells were seeded into the upper chamber. Next, 500 μL of RPMI 1640 medium containing 10% fetal calf serum (FCS) was added into the lower well. After incubation for 4 hours at 37°C, the migrated cells were counted by flow cytometry for 60 seconds. A sample of non‐migrated cells served as a reference.

### 2.9 CellTrace cell proliferation assay

Cell labeling with CellTrace Violet was performed according to the protocols provided by the manufacturer (CellTrace Violet Cell Proliferation Kit, Invitrogen, Molecular Probes). Human AML cells were stained with CellTrace Violet and cultured at 1 × 10^6^ cells/well. Cell proliferation was then determined via flow cytometry (LSR II, BD Biosciences) on day 4, and the data were analyzed by ModFit software (Verity Software House, USA).

### 2.10 Ki67 analysis

For Ki67 analysis, cells were fixed and permeabilized using the reagent A/B solutions provided in the BD IntraSure^™^ kit (BD Biosciences) according to the kit instructions. Briefly, cells were incubated with reagent A for 5 minutes at room temperature (RT). Then, the cells were treated with hemolysis solution (BD Biosciences) and centrifuged. The cell pellets were washed with PBS and suspended in 50 μL of reagent B, and then stained with 5 μL of FITC‐conjugated anti‐Ki67 antibody (eBioscience) for 30 minutes in the dark at RT. The cells were then washed with PBS and stained with Hoechst 33342 (Sigma). Cell cycle progression was assessed using a FACScan cytometer (Canto II, BD Biosciences) and the data were analyzed by FlowJo software.

### 2.11 Apoptosis assay

For apoptosis detection, cells were collected and stained with APC‐annexin V and 7‐AAD (BD Pharmingen^™^, San Jose, CA, USA) following the manufacturer's instructions. Annexin V‐positive cells were identified by flow cytometry (LSR II, BD Biosciences), and the data were analyzed by FlowJo software.

### 2.12 Quantitative real‐time PCR analysis

For the qRT‐PCR assay, total RNA was extracted using the RNeasy Mini Kit (QIAGEN) according to the manufacturer's protocol. cDNA was synthesized using a TransScript First‐Strand cDNA Synthesis SuperMix Kit (TransGen Biotech, Beijing, China) according to the manufacturer's instructions. qRT‐PCR assays were performed using SYBR Green (Roche) with a Step One Plus Real‐Time PCR System (Applied Biosystems) to detect the expression levels of the indicated genes. The cycle numbers of the target genes were normalized to that of the housekeeping gene GAPDH to obtain a ΔCT value. The calculation equation is 2^−ΔΔCT^. The primer sequences used to amplify the indicated genes were as follows: GACAGTCAGCCGCATCTTCT (GAPDH forward), TTAAAAGCAGCCCTGGTGAC (GAPDH reverse), GGGAGGTTCGTGGGAGTAAG (PRDM5 forward), and TCAGAAGCTCCGTGTCTGTTT (PRDM5 reverse).

### 2.13 Western blot analysis

Cells were lysed by RIPA buffer (Beyotime, Shanghai, China) supplemented with protease inhibitor cocktail and PMSF (Roche, USA). Total protein was extracted and subjected to Western blotting. Equal amounts of total protein were separated by SDS‐PAGE and transferred to PVDF membranes (Millipore, Bedford, MA). The immunoreactive bands were blocked with 5% bovine serum albumin for 1 hour and incubated with primary antibodies overnight at 4°C. After three washes with TBST, the membranes were incubated with HRP‐conjugated secondary antibodies for 1 hour at RT and were then washed with TBST an additional 3 times. Protein bands were detected by using enhanced chemiluminescence Western blotting detection reagents (Pierce, USA). Images were acquired for further analysis.

### 2.14 Tumor xenografts in nude mice

All animal experiments were performed in compliance with the Guide for the Care and Treatment of Laboratory Animals of Tongji Hospital. The in vivo tumorigenicity of OCI‐AML3 and U937 cells (control [CT] vs overexpressing [OE]) cells was tested in athymic nude mice. Female nude mice were injected subcutaneously in the left and right dorsal flank with a suspension of 5 × 10^6^ OCI‐AML3 CT and OE cells, respectively, in serum‐free medium (0.1 mL). As for U937 xenograft tumor, female nude mice were inoculated subcutaneously in the right flank with 2.5 × 10^6^ U937 CT or OE cells. Tumor volumes (πLW^2^/6) were estimated using digital calipers every other day after injection. All mice were kept in a pathogen‐free animal facility at Tongji Hospital of Tongji Medical College, Huazhong University of Science and Technology, Wuhan, China, and were sacrificed by cervical dislocation 12 days after inoculation.

### 2.15 Histology and immunohistochemistry

The tumor tissues were fixed in formalin, embedded in paraffin and sectioned for further hematoxylin and eosin (HE) staining and immunohistochemistry (IHC). Tissue sections were deparaffinized in xylene and rehydrated in a series of graded alcohols and distilled water. Slides were processed for antigen retrieval by a standard microwave heating technique. Specimens were incubated with anti‐Ki67 (ab15580, Abcam) and PRDM5 (NOVUS) primary antibodies overnight at 4°C. Antibody detection was performed using DAB according to the manufacturer's protocol. Images of typical sections were acquired using an OLYMPUS microscope (Tokyo, Japan).

### 2.16 Statistical analysis

GraphPad Prism 7.0 software was used for statistical analysis. The data were expressed as the means ± SDs of 3 independent experiments. Significant differences between two groups were statistically analyzed using Student's *t* test. One‐way ANOVA was applied to analyze the differences among more than two groups. A log‐rank (Mantel‐Cox) test was used to determine the *P* values for all Kaplan‐Meier survival analyses. In all cases, *P* < 0.05 was considered statistically significant. Significance is denoted as *P < 0.05, **P < 0.01, ***P < 0.001, and ****P < 0.0001.

## 3 RESULTS

### 3.1 A high level of PRDM5 in AML is associated with poor prognosis and survival

**Figure 1:**
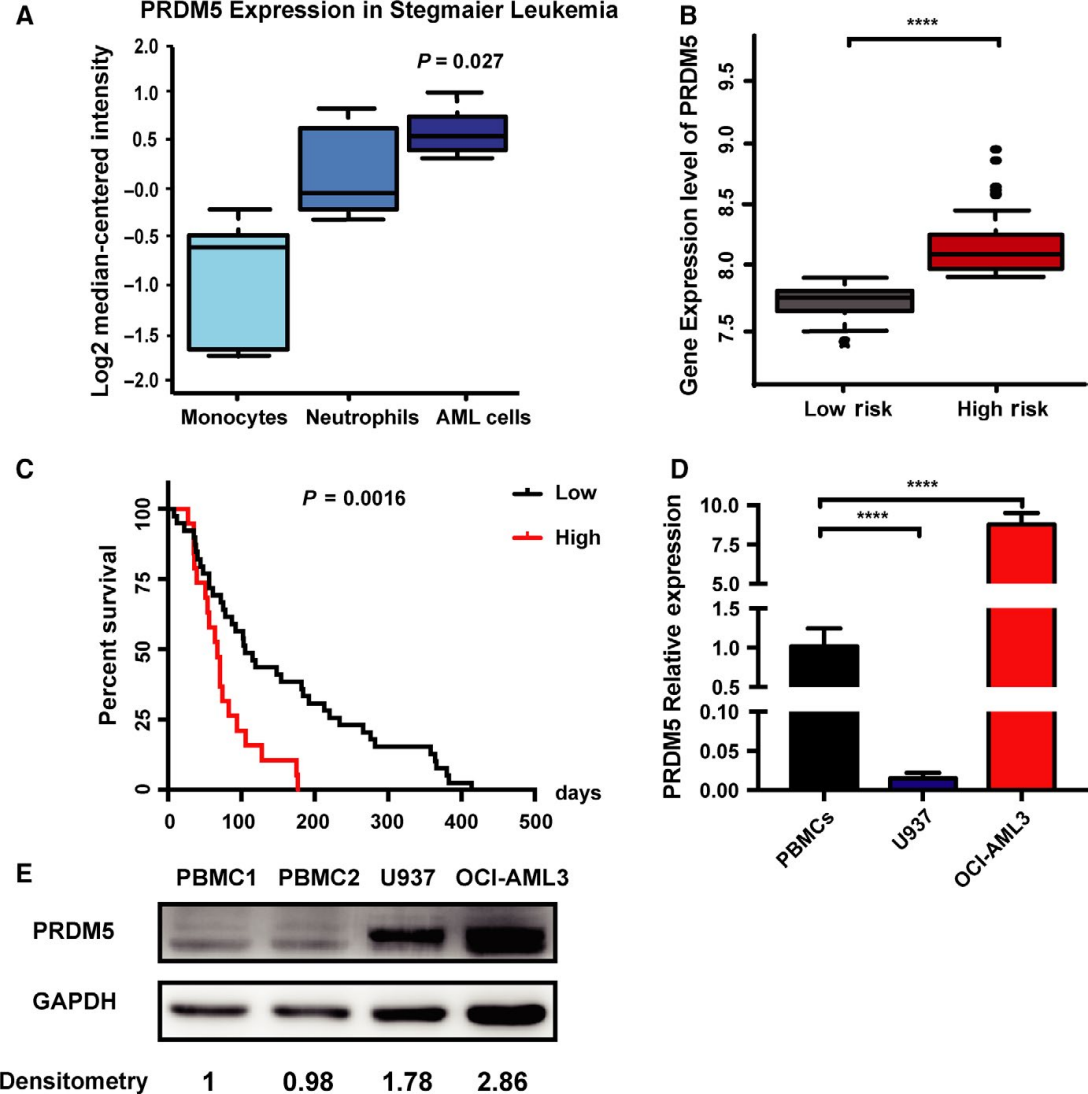
Upregulation of PRDM5 in AML is associated with poor prognosis and survival. A, PRDM5 mRNA expression in monocytes, neutrophils, and AML cells was analyzed in Stegmaier leukemia (from the Oncomine database). B, Box plot showing the PRDM5 gene expression level by risk groups in the AML GSE12417‐GPL96 dataset generated by Metzeler and Buske (from the SurvExpress database). C, The Kaplan‐Meier curve comparing the PRDM5 high‐expression population (red) and PRDM5 low‐expression population (black) of AML patients was created from the PrognoScan database. D, Expression level of PRDM5 in PBMCs from 3 healthy donors and in two human AML cell lines (OCI‐AML3 and U937). E, Western blotting analysis of protein level of PRDM5 in PBMCs, OCI‐AML3, and U937 cells. ****P < 0.0001, by Student's *t* test. AML, acute myeloid leukemia; PBMCs, peripheral blood mononuclear cells

To determine the mRNA expression of PRDM5 in AML patients and normal donors, we analyzed microarray data from AML patients with Stegmaier leukemia (available through https://www.oncomine.org). PRDM5 expression was slightly upregulated in AML cells compared to that in monocytes and neutrophils (Figure 1A). To clarify the prognostic value of PRDM5 gene expression in AML, we performed a large cohort survival analysis on data from two public databases with transcriptome analysis results. Through the SurvExpress program (http://bioinformatica.mty.itesm.mx/SurvExpress), patients in the AML GSE12417‐GPL96 dataset generated by Metzeler and Buske were divided into “Low Risk” and “High Risk” groups according to the prognostic index. Survival analysis of the RNA microarray database predicted that the “High Risk” group of patients with AML presented a significantly higher PRDM5 expression level and had a poorer prognosis than the “Low Risk” group (Figure 1B). Using the publicly available microarray datasets from PrognoScan (http://www.abren.net/PrognoScan/), we found that higher expression of PRDM5 predicted significantly lower OS in AML patients (Figure 1C). We next examined the mRNA level of PRDM5 in PBMCs from 3 healthy donors and in 2 human AML cell lines (OCI‐AML3 and U937). In accordance with the results from the public microarray dataset, PRDM5 expression in the OCI‐AML3 cell line was significantly higher than that in normal PBMCs, while in the U937 cell line, the expression was relatively lower (Figure 1D). We also measured the protein levels of PRDM5 in these cells and found that both U937 cells and OCI‐AML3 cells had higher protein level of PRDM5 than that of PBMCs, although the PRDM5 protein level in U937 cells was relatively lower than that in OCI‐AML3 cells (Figure 1E). The data showed that AML patients with higher expression of PRDM5 had poorer prognoses and survival outcomes than those with lower expression of PRDM5.

### 3.2 PRDM5 overexpression promotes OCI‐AML3 and U937 cell growth and migration in vitro

**Figure 2:**
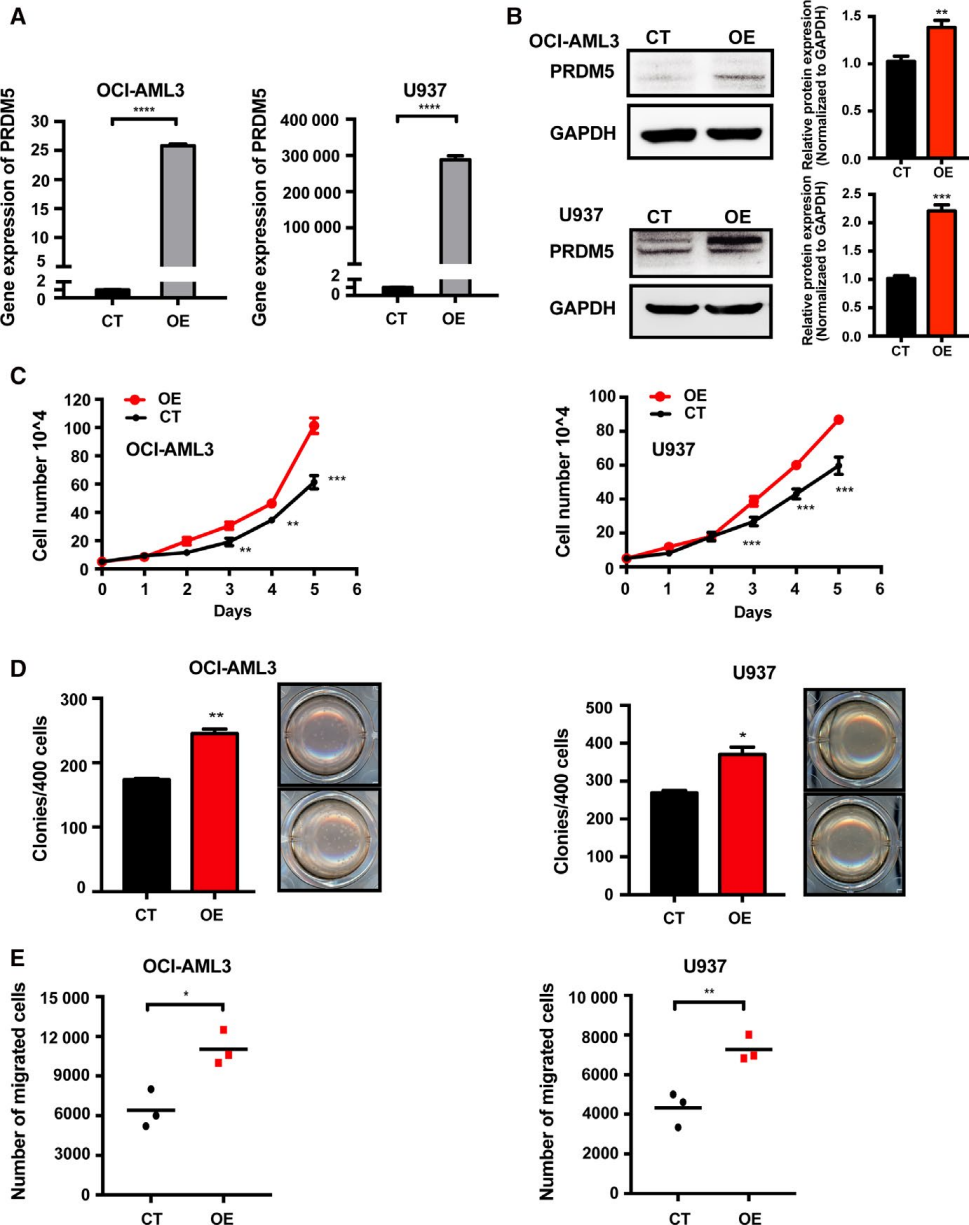
PRDM5 overexpression promotes the growth and migration of OCI‐AML3 and U937 AML cells. A, Real‐time PCR confirmed the PRDM5 expression level in lentivirus‐transduced empty Migr1 (CT) and PRDM5‐mCherry (OE) OCI‐AML3 and U937 cells. B, Western blot analysis verified the upregulation of PRDM5 in OCI‐AML3 and U937 OE cells. C, The effect of PRDM5 on the growth of OCI‐AML3 and U937 CT and OE cells was assessed using the viable cell counting assay over 6 consecutive d. D, The colony formation assay was performed with OCI‐AML3 and U937 CT and OE cells. E, The transwell assay showed that the overexpression of PRDM5 increased the migratory ability of OCI‐AML3 and U937 cells. *P < 0.05, **P < 0.01, ***P < 0.001, ****P < 0.0001, by Student's *t* test. CT, control; OE, overexpressing

To explore the role of PRDM5 in AML with high and low PRDM5 expression, we next assessed the effects of PRDM5 in OCI‐AML3 and U937 cell lines using a lentiviral system to overexpress PRDM5‐mCherry. OCI‐AML3 and U937 cells transduced with PRDM5‐mCherry (referred to as OCI OE and U937 OE) showed significantly higher expression of PRDM5 mRNA and protein than the corresponding cells transduced with empty Migr1 retrovirus as a control (referred to as OCI CT and U937 CT) (Figure 2A,B). Then, we counted the number of viable cells for 6 consecutive days and found that cells OE PRDM5 grew much faster than the control cells (Figure 2C). In the colony formation assay, overexpression of PRDM5 significantly increased colony formation ability when compared with that observed in OCI‐AML3 and U937 control cells (Figure 2D). Moreover, the migration ability of OCI OE and U937 OE cells was appreciably greater than that of the corresponding CT cells (Figure 2E). These results indicated that PRDM5 increases cell growth, colony formation, and migration abilities in AML cell lines.

### 3.3 PRDM5 overexpression enhances OCI‐AML3 and U937 cell proliferation and cell cycle progression without affecting apoptosis

**Figure 3:**
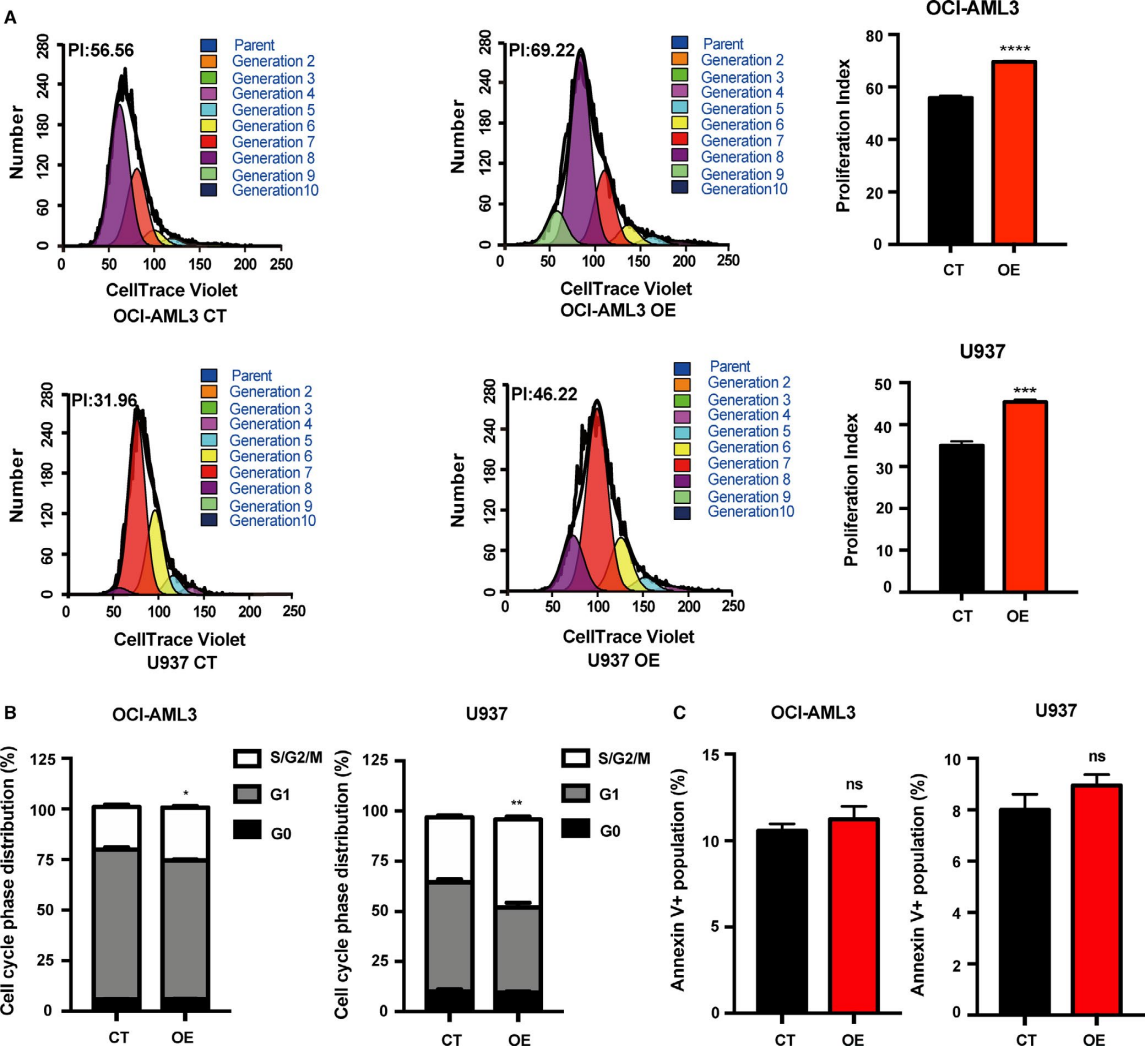
Overexpression of PRDM5 enhances OCI‐AML3 and U937 cells proliferation and cell cycle progression without affecting apoptosis. A, The CT and OE AML cells were stained with CellTrace Violet and cultured for 4 d. The number of cells in each generation was estimated by deconvolution of the FACS data, and the proliferation index was calculated using ModiFit software. B, Cell cycle analysis of the CT and OE cells was performed by Ki67 and Hoechst 33342 staining. The graph showed the relative cell cycle distribution of OCI‐AML3 and U937 CT and OE cells. C, Flow cytometric analysis of the apoptosis of OCI‐AML3 and U937 CT and OE cells. The graph showed the quantification of apoptotic cells as a percentage of annexin V‐positive cells. *P < 0.05, **P < 0.01, ***P < 0.001, ****P < 0.0001, ns: no significant difference, by Student's *t* test. AML, acute myeloid leukemia; CT, control; OE, overexpressing

Given that PRDM5 potentially plays an important role in AML, we next explored the effects of PRDM5 on proliferation, cell cycle, and apoptosis using flow cytometric analysis. The CellTrace cell proliferation assays showed that the proliferative capacity of PRDM5‐OE cells was modestly elevated (Figure 3A). Then, we analyzed cell cycle changes by Ki67 and Hoechst staining to differentiate cells in the G0, G1, and S/G2/M phases. The number of OE cells in the S/G2/M phases was higher than that of CT cells, while the number of OE cells in the G1 phases was relatively decreased (Figure 3B). However, as measured by flow cytometry with annexin‐V/7‐AAD staining, the percentage of apoptotic OE cells and CT cells was similar (Figure 3C). Taken together, these data suggested that overexpression of PRDM5 promotes AML cell proliferation and cell cycle progression.

### 3.4 PRDM5‐mediated progression of OCI‐AML3 and U937 cells is partially regulated through JNK pathway activation

**Figure 4:**
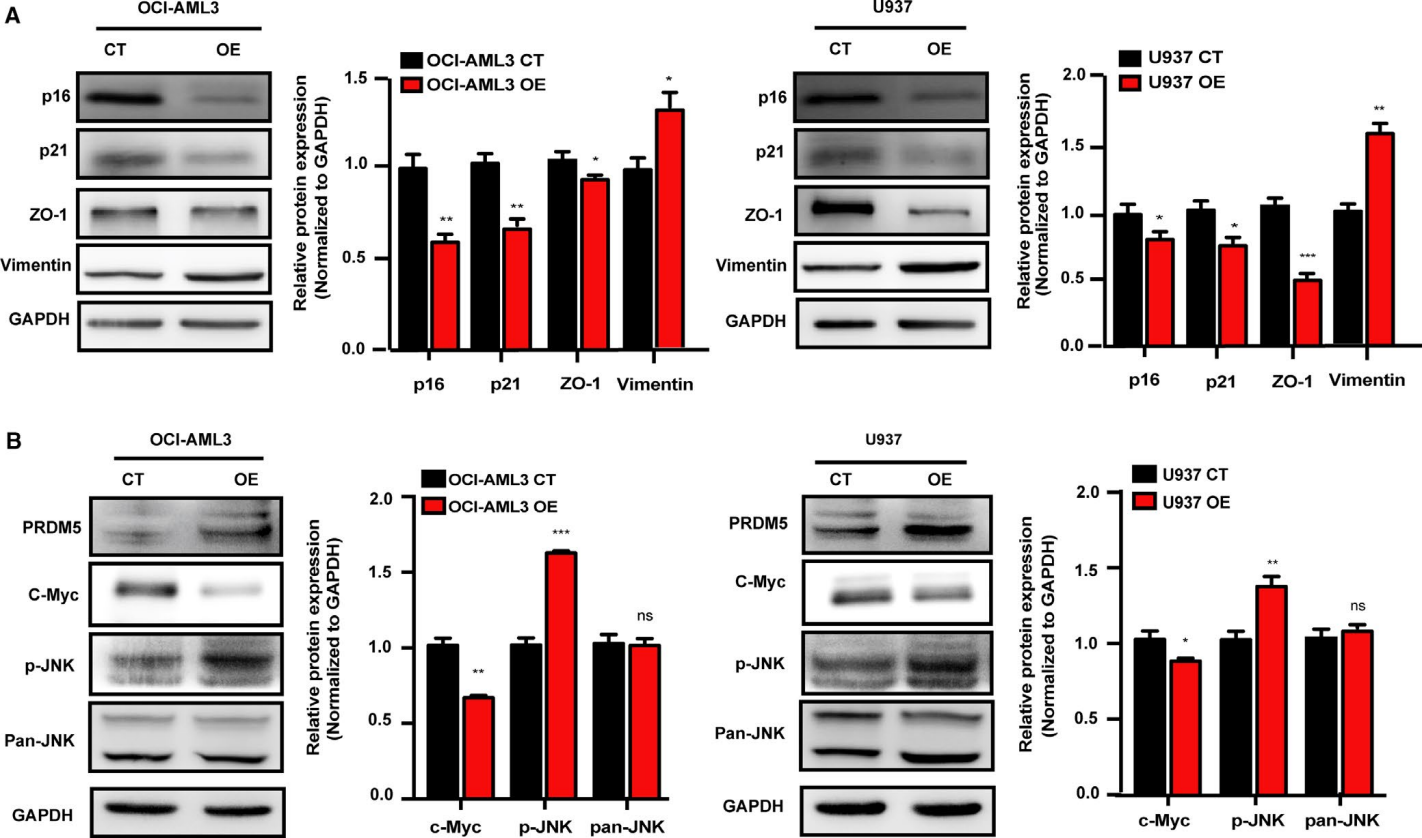
PRDM5 induces AML cells proliferation and migration through promoting cell cycle progression and EMT accompanied with JNK pathway activation and c‐Myc downregulation. Western blotting analysis of total protein lysates from OCI‐AML3 and U937 CT or OE cells. p16, p21, ZO‐1, and Vimentin protein levels (A) and the protein levels of JNK pathway and c‐Myc (B) were measured. The bar plots indicated the relative quantification of each band intensity for the OE cells (normalized to that of GAPDH) compared with that of the corresponding CT cells. Each experiment was repeated 3 times. *P < 0.05, **P < 0.01, ***P < 0.001, ns: no significant difference, by Student's *t* test. AML, acute myeloid leukemia; CT, control; EMT, epithelial‐mesenchymal transition; JNK, Jun N‐terminal kinase; OE, overexpressing

Tumorgenicity is determined by tumor growth and its metastasis ability. Cell cycle regulators p16 and p21 have been shown to inhibit tumor cell proliferation and induce cell cycle arrest^21^ while epithelial‐mesenchymal transition (EMT) in metastasis is characterized by the decrease of epithelial proteins expression (E‐cadherin, ZO‐1 and claudin‐1) and the increase of mesenchymal proteins such as Vimentin and Fibronectin.^22^ Here, in our study, we found that the protein levels of p16 and p21 in PRDM5 overexpressed AML cells were decreased. PRDM5 overexpression also promoted Vimentin expression and inhibited ZO‐1 expression (Figure 4A). The increase in JNK phosphorylation has been shown to be an oncogenic event in the growth and migration of multiple cancers.^15^, ^23^ To determine the signaling pathways involved in PRDM5‐mediated progression of OCI‐AML3 and U937 cells, we next focused on the JNK pathway. Our results showed that JNK activation significantly increased in cells OE PRDM5 compared with that in control cells, while the total protein levels were not changed. c‐Myc is a well‐known protooncogene in many human cancers and its activation promotes cell motility and migration. Surprisingly, we found that the expression level of c‐Myc was downregulated in OE cells (Figure 4B).

**Figure 5:**
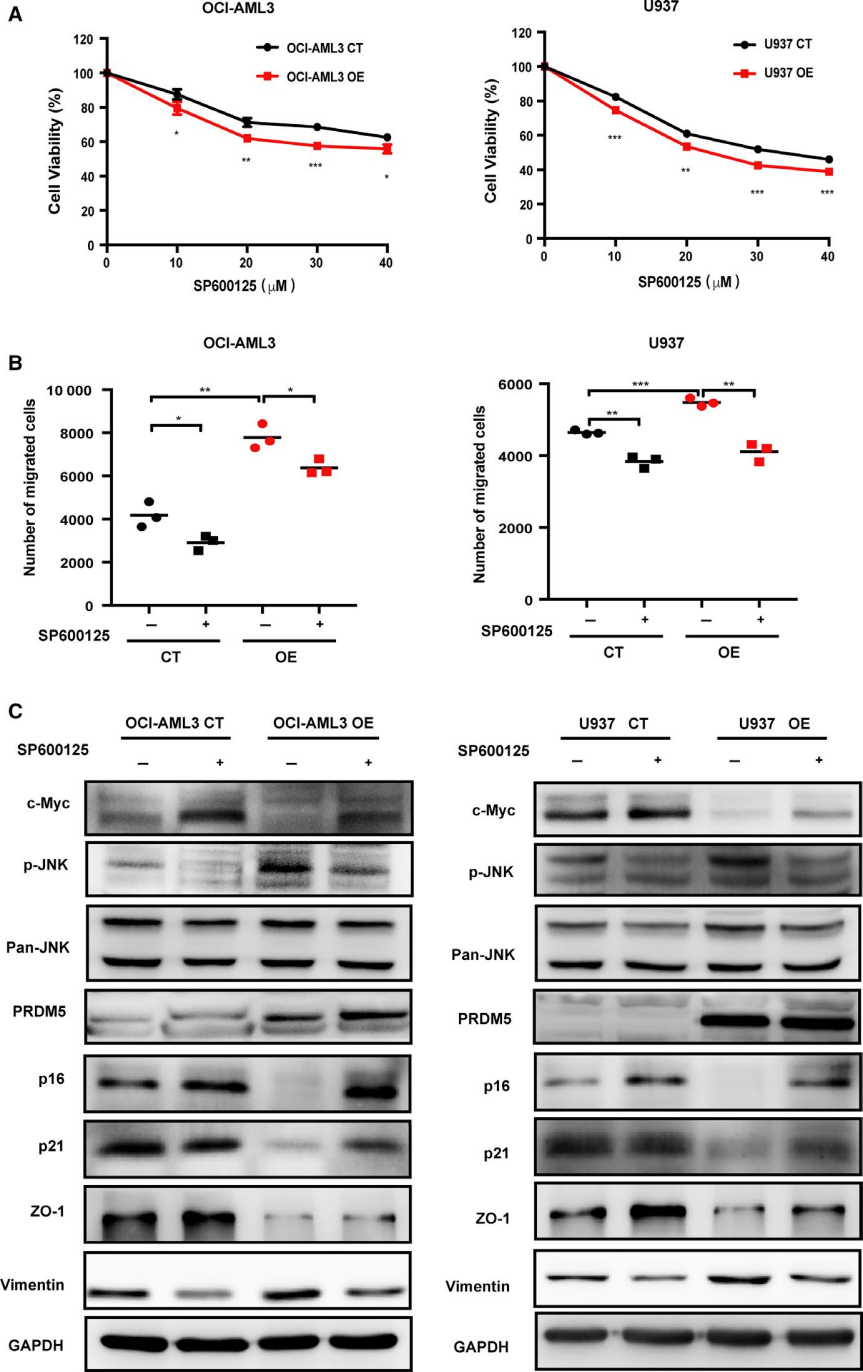
The JNK inhibitor SP600125 inhibits the JNK signaling pathway and rescues the effects of PRDM5 overexpression in OCI‐AML3 and U937 cells. A, Cells were incubated with indicated concentrations of SP600125 for 24 h, and a CCK‐8 assay was performed to determine the viability of OCI‐AML3 and U937 CT and OE cells. B, A transwell assay was carried out with OCI‐AML3 and U937 CT and OE cells treated with or without 20 μmol/L SP600125. C, c‐Myc, p‐JNK, Pan‐JNK, PRDM5, p16, p21, ZO‐1, Vimentin, and GAPDH expression was detected by Western blotting in OCI‐AML3 and U937 CT and OE cells treated with or without 20 μmol/L SP600125. *P < 0.05, **P < 0.01, ***P < 0.001 by one‐way ANOVA. CT, control; JNK, Jun N‐terminal kinase; OE, overexpressing

To confirm that JNK activation was responsible for PRDM5‐mediated proliferation and migration of OCI‐AML3 and U937 cells, we used the specific JNK inhibitor SP600125 to inhibit the JNK signaling pathway. As expected, SP600125 induced a dose‐dependent reduction in cell viability in the above leukemia cells. In addition, PRDM5‐OE cells appeared much more sensitive to SP600125 than CT cells (Figure 5A). The transwell assay also confirmed that SP600125 blocked PRDM5‐induced migration (Figure 5B). Notably, Western blotting analysis confirmed that JNK activation was partially inhibited by SP600125. Interestingly, the protein expression of c‐Myc was upregulated in both SP600125‐treated CT and OE cells. Furthermore, we found that the protein levels of p16, p21, and ZO‐1 were elevated whereas the level of Vimentin was reduced in SP600125 treated PRDM5‐OE cells (Figure 5C). These findings indicated that JNK signaling pathway is involved in the PRDM5‐induced proliferation and migration of OCI‐AML3 and U937 cells.

### 3.5 PRDM5 overexpression accelerates the in vivo growth of OCI‐AML3 and U937 human leukemic xenograft tumors

**Figure 6:**
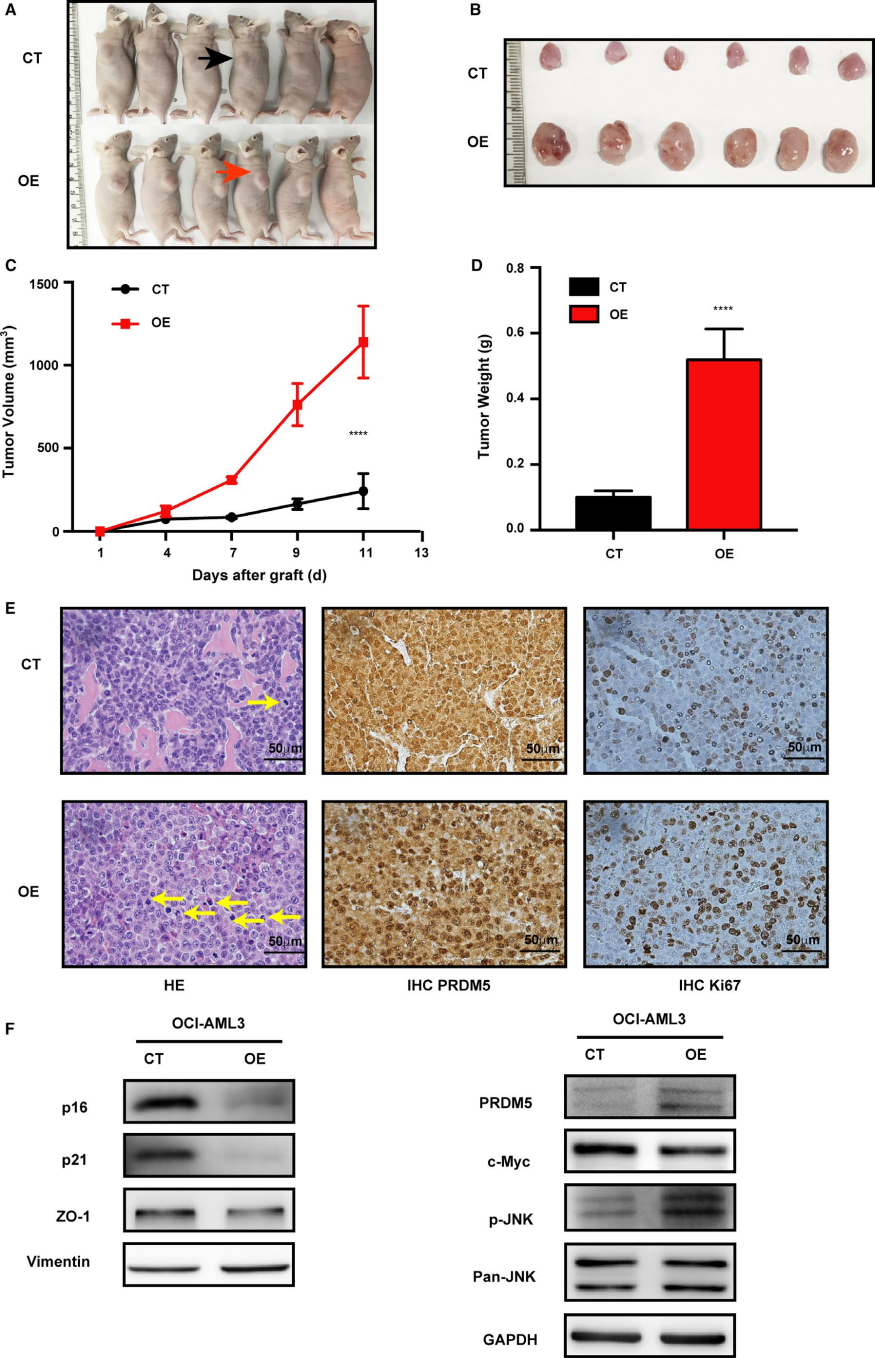
PRDM5 overexpression accelerates the growth of OCI‐AML leukemic xenograft tumors. A, Representative nude mice image showed the CT tumor on the left flank and the PRDM5 OE tumor on the right flank. B, Photographs of tumor masses were collected at the end of the experiment. C, Growth curves of tumors generated by OCI‐AML3 CT and OE cells in vivo. D, Weight of subcutaneously formed tumors. E, HE staining (left panel) and immunohistochemical analysis of PRDM5 (middle panel) and Ki67 (right panel) expressions were shown in the xenograft tumors from each group. F, Western blotting analysis of related protein expression in tumor cells of CT and OE groups. ****P < 0.0001, by Student's *t* test. AML, acute myeloid leukemia; CT, control; HE, hematoxylin and eosin; OE, overexpressing

Due to the distinct proliferation‐promoting effects of PRDM5 overexpression in leukemia cells observed in vitro, we then examined whether overexpression of PRDM5 affects the oncogenic capacity of AML cells in vivo. OCI‐AML3 cells transfected with PRDM5 (OE) and Migr1 (CT) lentivirus were subcutaneously inoculated into nude mice. Cells transduced with PRDM5 lentivirus grew rapidly in nude mice. In contrast, cells transduced with Migr1 lentivirus resulted in slow‐growing subcutaneous tumors (Figure 6A,B). Moreover, the inspection of gross tumor morphology showed a significant increase in the size and volume of tumors in PRDM5 OE group. HE staining of the tumor samples confirmed the increased expansion and pathologic mitosis of leukemia cells in OE group. Furthermore, IHC revealed a higher level of PRDM5 and Ki67 in the tumor tissues of PRDM5 OE group than in those of CT group. Western blotting analysis of whole cell lysates of subcutaneous tumors showed that the protein level of p16, p19, ZO‐1 was down‐regulated while the expression of Vimentin was upregulated in PRDM5 OE group. More importantly, the JNK pathway was activated and c‐Myc was decreased in PRDM5 OE group. Additionally, we also performed a similar experiment to investigate the oncogenic capacity of U937 CT and OE cells in vivo. The tumorigenic ability of U937 OE cells is higher than that of U937 CT cells in vivo (Figure S1), consistent with those in OCI‐AML cells. Taken together, our study suggested that overexpression of PRDM5 facilitates the growth of OCI‐AML3 and U937 xenograft tumors.

## 4 DISCUSSION

PRDM5 is a member of the PRDM family and acts as a sequence‐specific DNA‐binding transcriptional suppressor or activator that targets various proteins involved in development, cell cycle, tumor suppression, and cell adhesion.^24^ PRDM5 is generally implicated as a tumor suppressor in a variety of cancers due to the hypermethylation of its promoter.^25^, ^26^ However, a recent study by Wang et al showed that PRDM5 acts as an important tumor promoter in the progression of malignant melanoma.^14^ Although PRDM5 has currently been studied in many cancers, the exact role and regulatory mechanism of PRDM5 in cancers are not completely understood. In our study, we observed that a high expression level of PRDM5 was associated with poor prognosis in AML patients. PRDM5 protein overexpression drove AML progression by significantly enhancing the tumor growth, proliferation, and migration in vitro. These findings contrast with those of previous studies that overexpression of PRDM5 resulted in the suppression of cell proliferation through regulating either G2M arrest or apoptosis.^10^, ^24^ Interestingly, we demonstrated that PRDM5 promoted cell cycle progression without affecting apoptosis in OCI‐AML3 and U937 cells. Moreover, we revealed that PRDM5 overexpression dramatically promoted leukemia cell growth and proliferation in vivo. The combined in vitro and in vivo studies strongly suggested that PRDM5 functioned as an oncogene in AML. Interestingly, although we found that the endogenous mRNA expression of PRDM5 was much higher in OCI‐AML3 cells than the normal PBMCs while PRDM5 expression was much lower in U937 cells than the normal PBMCs, the PRDM5 protein levels in U937 and OCI‐AML3 cells were both higher than that of the PBMCs, suggesting high level of PRDM5 promotes tumorigenesis. We speculated that the discrepancy of mRNA and protein expression level of PRDM5 in U937 cells may be due to the post‐transcriptional or post‐translational modifications.

The loss of proliferation inhibition and acquisition of advanced migration ability are common features of cancer cells. JNK and p38 MAPK family members integrate signals that affect proliferation, differentiation, survival and migration in tumorigenesis.^15^ Increased JNK activation has been demonstrated in many studies of human cancers, including glioma, prostate carcinoma, osteosarcoma, and squamous cell carcinoma.^27^ The JNK pathway is a three‐tiered cascade. After activation, MAPKs phosphorylate specific serine and threonine residues on target substrates that in turn promotes tumor cell proliferation, migration and invasion.^14^, ^15^ It has been reported that the JNK pathway activation promotes the proliferation of cancer cells by reducing the expression level of cell cycle inhibitors p16 or p21.^27^, ^28^ What's more, the JNK pathway could accelerate cancer cells migration through inducing the EMT which is accompanied with the changed expression of related proteins such as ZO‐1 and Vimentin.^29^ EMT‐like processes have been implicated in hematological malignancies in addition to solid tumors,^30^, ^31^ while p38/JNK pathway was shown to be involved in portulacerebroside A‐mediated suppression of adhesion, migration and invasion in AML cell lines.^32^ To investigate the association of PRDM5 and the JNK signaling pathway in AML, we first confirmed that the overexpression of PRDM5 was followed by downregulated expression of p16, p21, ZO‐1, and upregulated expression of Vimentin in OCI‐AML3 and U937 cells. We then verified that PRDM5‐OE AML cells had an increased level of phosphorylated JNK but not their total JNK protein level. More importantly, a JNK inhibitor SP600125 blocked JNK activity, reversed the increase in the proliferation and migration of PRDM5‐OE cells with the increased expression of p16, p21, and ZO‐1, and decreased the expression of Vimentin. All these results demonstrated that PRDM5 induced the proliferation and migration of AML cells partially through regulating the JNK pathway. Interestingly, we observed that although c‐Myc expression was downregulated in OE cells, the JNK inhibitor increased the expression of c‐Myc. c‐Myc has been shown to be a protooncogene that modulates numerous cancer cell behaviors, including proliferation, differentiation, migration, and genomic instability.^33^, ^34^, ^35^ Recently, Ma et al demonstrated that c‐Myc also suppressed cell migration in Drosophila and human lung adenocarcinoma cell lines in a JNK‐dependent manner.^36^ Consistent with the results of that research, our results indicated that suppressed JNK activity was positively correlated with c‐Myc expression. However, further studies need to be conducted to investigate the specific relationship between JNK activity and c‐Myc function.

**Figure 7:**
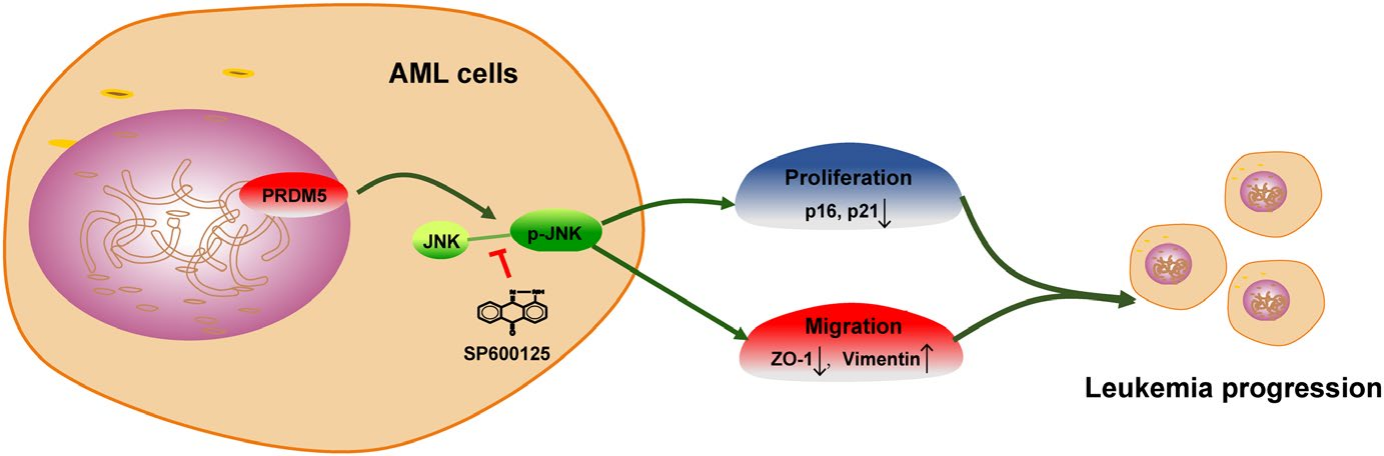
Schematic diagram summarizes the role of PRDM5 in AML progression. Overexpression of PRDM5 promotes AML cell proliferation and migration partially via JNK pathway activation. AML, acute myeloid leukemia; JNK, Jun N‐terminal kinase

In summary, we demonstrate in this study that overexpression of PRDM5 promotes AML cell proliferation and migration partially via JNK activation (Figure 7). PRDM5 overexpression thus could act as a pro‐carcinogenic factor in AML. Therefore, PRDM5 could potentially be an attractive therapeutic candidate for AML patients.

## CONFLICT OF INTEREST

The authors declare no potential conflicts of interest.

## Data Availability

I confirm that I have included a citation for available data in my references section, unless my article type is exempt.
