# Supplementary material for: Overexpression of PRDM5 promotes acute myeloid leukemia cell proliferation and migration by activating the JNK pathway
Source: Cancer Med. 2019 May 23;8(8):3905–17. doi: 10.1002/cam4.2261 (PMC6639193; doi:10.1002/cam4.2261)
Supplement: Supplementary file 1 [file CAM4-8-3905-s001.pdf]

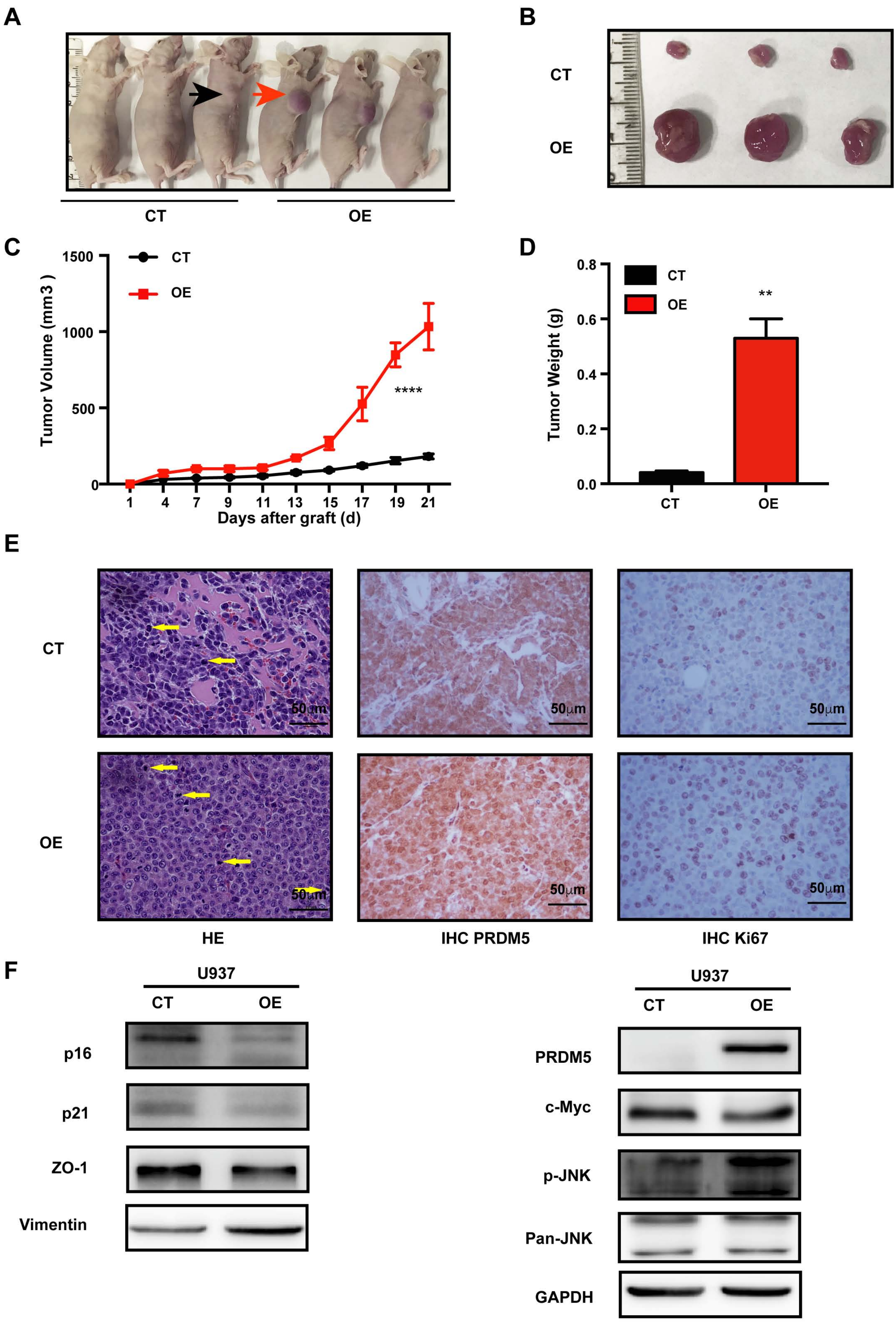


**Supplementary Figure 1. PRDM5 overexpression enhances the tumorigenic ability of U937 cells in vivo. (A-B) Photographs of representative tumors in U937 CT and OE Group. (C) Tumor growth in nude mice bearing subcutaneous U937 CT and OE xenografts. (D) Weight of subcutaneously formed tumors. (E) Representative images of HE staining (left panel) and immunohistochemistry for PRDM5 (middle panel) and Ki67 (right panel) expression are shown in the xenograft tumors from each group. (F) p16, p21, ZO-1, Vimentin, PRDM5, c-Myc, p-JNK and Pan-JNK protein expression were determined by western blotting analysis in CT and OE groups. **P<0.01, ****P<0.0001,by Student's t-test.**
